# Supplementary material for: Risk factors for depression in patients with Parkinson’s disease: A nationwide nested case-control study
Source: PLoS One. 2020 Jul 27;15(7):e0236443. doi: 10.1371/journal.pone.0236443 (PMC7384643; doi:10.1371/journal.pone.0236443)
Supplement: S1 Table — (DOC) [file pone.0236443.s001.doc]

| **S1 Table. ICD-9-CM and ATC codes used in this study.** | |
| --- | --- |
| **Main diseases** | **ICD-9-CM codes** |
| Parkinson’s disease | 332 |
| Dementia | 290, 294.1, 331.0 |
| Stroke | 430-434, 436-438 |
| Depression | 296.2-296.3, 296.82, 300.4, 311 |
| Psychosis | 295, 297 |
| **Comorbidities** | **ICD-9-CM codes and ATC codes** |
| Diabetes mellitus | 249-250, 648.01, 648.02, 588.1, 357.2 |
| Hypertension | 401 |
| Chronic pulmonary disease | 416.8, 416.9, 490, 491-495, 496, 500-505, 506.4, 508.1 |
| Osteoporosis | 733.0 |
| Chronic heart failure | 402.01, 402.11, 402.91, 404.01, 404.03, 404.11, 404.13, 404.91, 404.93, 425.4, 425.9, 428.4 |
| Chronic kidney disease | 581-583, 585-587 |
| Chronic liver disease | 571.40, 571.41, 571.49, 571.2, 571.5, 571.6, 572.2, |
| Cancers | 140-208 |
| Anxiety disorders | 300.00-300.02, 300.09, 300.2, 300.3, 308, 309.81, 293.84 |
| Sleep disturbance | 780.5, 307.4 |
| **Drug categories** | **ATC codes** |
| Anti-PD Medications |  |
| Levodopa | N04BA01 |
| Levodopa and decarboxylase inhibitor | N04BA02 |
| Levodopa, decarboxylase inhibitor and COMT inhibitor | N04BA03 |
| Entacapone | N04BX02 |
| Bromocriptine mesylate | N04BC01 |
| Pergolide mesylate | N04BC02 |
| Cabergoline | N04BC06 |
| Ropinirole | N04BC04 |
| Pramipexole | N04BC05 |
| Amantadine | N04BB01 |
| Selegilline | N04BD01 |
| Hypnotic Medications |  |
| Clonazepam | N03AE01 |
| Diazepam | N05BA01 |
| Chlordiazepoxide | N05BA02 |
| Medazepam | N05BA03 |
| Oxazepam | N05BA04 |
| Potassium clorazepate | N05BA05 |
| Lorazepam | N05BA06 |
| Adinazolam | N05BA07 |
| Bromazepam | N05BA08 |
| Clobazam | N05BA09 |
| Ketazolam | N05BA10 |
| Prazepam | N05BA1 |
| Alprazolam | N05BA12 |
| Halazepam | N05BA13 |
| Pinazepam | N05BA14 |
| Camazepam | N05BA15 |
| Nordazepam | N05BA16 |
| Fludiazepam | N05BA17 |
| Ethyl loflazepate | N05BA18 |
| Etizolam | N05BA19 |
| Clotizepam | N05BA21 |
| Cloxazolam | N05BA22 |
| Tofisopam | N05BA23 |
| Lorazepam, combinations | N05BA56 |
| Flurazepam | N05CD01 |
| Nitrazepam | N05CD02 |
| Flunitrazepam | N05CD03 |
| Estazolam | N05CD04 |
| Triazolam | N05CD05 |
| Lormetazepam | N05CD06 |
| Temazepam | N05CD07 |
| Midazolam | N05CD08 |
| Brotizolam | N05CD09 |
| Quazepam | N05CD10 |
| Loprazolam | N05CD11 |
| Doxefazepam | N05CD12 |
| Cinolazepam | N05CD13 |
| Zopiclone | N05CF01 |
| Zolpidem | N05CF02 |
| Zaleplon | N05CF03 |
| Eszopliclone | N05CF04 |
| Abbreviations: ATC, Anatomical Therapeutic Chemical; COMT, catechol-O-methyltransferase; ICD-9-CM, International classification of diseases, ninth revision, clinical modification; PD, Parkinson’s disease. | |
